# The association between antibody levels before and after 7-valent pneumococcal conjugate vaccine immunization and subsequent pneumococcal infection in chronic arthritis patients

**DOI:** 10.1186/s13075-015-0636-z

**Published:** 2015-05-19

**Authors:** Johanna Nagel, Pierre Geborek, Tore Saxne, Göran Jönsson, Martin Englund, Ingemar F Petersson, Jan-Åke Nilsson, Lennart Truedsson, Meliha C Kapetanovic

**Affiliations:** Department of Clinical Sciences, Lund, Section of Rheumatology, Lund University, and Skåne University Hospital, Kioskgatan 3, SE-221 85 Lund, Sweden; Department of Clinical Sciences Lund, Section of Infectious Diseases, Lund University and Skåne University Hospital, Kioskgatan 3, SE-221 85 Lund, Sweden; Epidemiology and Register Centre South, Skåne University Hospital, Lund, and Orthopaedics, Department of Clinical Sciences Lund, Lund University, Barngatan 2B, Lund, SE-221 85 Sweden; Clinical Epidemiology Research & Training Unit, Boston University School of Medicine, 650 Albany Street, Boston, MA 02118 USA; Department of Laboratory Medicine, Section of Microbiology and Immunology, Lund University, Sölvegatan 23, Lund, SE-223 62 Sweden

## Abstract

**Introduction:**

The aim of present study is to inverstigate the association between antibody levels after vaccination with 7-valent pneumococcal conjugate vaccine (PCV7) and subsequent serious pneumococcal infections in rheumatoid arthritis (RA) and spondylarthropathy (SpA) patients.

**Methods:**

A cohort of 497 patients (RA = 248 and SpA = 249) received a single dose of PCV7. At vaccination, patients were treated with methotrexate (MTX; n = 85), anti-tumour necrosis factor (anti-TNF) + MTX (n = 169), anti-TNF monotherapy (n = 158) and non-steroidal anti-inflammatory drugs (NSAIDs)/analgesics (n = 85). Antibody levels of serotypes 6B and 23B were analyzed before and 4 to 6 weeks after vaccination using standard enzyme-linked immunosorbent assay (ELISA). Serious pneumococcal infections (pneumonia/lower respiratory tract infection, meningitis, sepsis, septic arthritis) occurring within 4.5 years after vaccination were identified in the Skåne Healthcare Register using the International Classification of Diseases, tenth revision (ICD-10) codes. The association between post-vaccination antibody levels and protection against infections and determination of protective cutoff levels was explored using receiver operating characteristic (ROC) curves. Predictors of infection were studied using regression analyses.

**Results:**

Eighteen infections were registered in 15 patients before vaccination and 27 infections in 23 patients after vaccination. Patients with serious infections after vaccination had significantly lower post-vaccination antibody titres for both 6B (*P* = 0.04) and 23 F (*P* = 0.04). Post-vaccination antibody levels of at least 1.29 mg/L and 1.01 mg/L for 6B and 23, respectively, were associated with better protection from serious infections. Higher age, concomitant prednisolone but not MTX or anti-TNF were associated with such infections.

**Conclusions:**

Patients with more robust antibody responses after vaccination with pneumococcal conjugate vaccine were less likely to suffer from serious infections. High age and prednisolone at vaccination were associated with putative serious pneumococcal infections in this cohort.

**Trial registration number:**

EudraCT EU 2007-006539-29 and NCT00828997. Registered 23 January 2009.

## Introduction

Vaccination is an appealing strategy when attempting to reduce the major global public health problem of pneumococcal disease [1,2]. Two pneumococcal vaccines are currently in clinical use. One of them contains 23 capsular polysaccharides (PPV) that primarily induce a B cell-dependent immune response. This type of vaccine prevents bacteraemia but does not efficiently protect the host against pneumococcal infection [3,4]. In 2000, a novel pneumococcal conjugate vaccine (PCV) was launched containing capsular polysaccharides derived from the seven most frequent pneumococcal serotypes causing pneumococcal disease in children <2 years of age. Conjugation of capsular polysaccharides with a highly immunogenic protein, that is a non-toxic diphtheria toxoid, induces a B and T cell response [5] resulting in mucosal immunity and thus effectively protects against vaccine serotypes that induce invasive pneumococcal disease, thereby at the same time reducing vaccine serotype carrier rates [6,7]. Pronounced herd immunity resulted in a decrease in invasive pneumococcal diseases in vaccinees and non-vaccinees as well as reduced antibiotic resistance rates. However, recent studies report that serotypes eradicated by the vaccine are being replaced by non-vaccine pneumococcal serotypes. This so-called ‘replacement’ might soon threaten the success of vaccine use [8]. Heptavalent pneumococcal conjugate vaccine has been shown to prevent recurrent pneumococcal infections in HIV-infected adults [9]. In a recent report from a randomised placebo-controlled trial, effectiveness of the 13-valent pneumococcal conjugate vaccine against community-acquired pneumonia in adults 65 years and older was shown [10]. Subsequently, the PCV has been approved for the immunisation of adults. Since 2012, the Centers for Disease Control and Prevention (CDC) Advisory Committee on Immunization Practice (ACIP) recommends immunisation with a single dose of 13-valent pneumococcal conjugate vaccine followed by a dose of PPV to all adults with immunocompromised conditions [11]. The 2014 updated recommendations include use of both vaccines to all adults ≥65 years of age [12].

The majority of patients with inflammatory arthritis such as rheumatoid arthritis (RA) or spondylarthropathy (SpA) (including ankylosing spondylitis, psoriatic arthritis or other spondylarthropathies) are commonly treated with immunosuppressive remedies and should, according to ACIPs recommendations, be immunised with pneumococcal conjugate vaccine. However, studies showing that the vaccine is efficacious in preventing disease caused by serotypes included in the vaccine in these patients are scarce [10,13].

Awaiting such studies on antibody response following vaccination is commonly used as a surrogate measure of vaccine efficacy. In the Finnish study on polysaccharide vaccine against *Haemophilus influenzae type b* (*Hib*), the efficacy of the vaccine was found to correlate with post-vaccination antibody levels of 1 mg/L [14]. Based on the protective response to *Hib* vaccine in children, antibody levels of ≥1 mg/L were estimated to be required for the long-term protection against encapsulated bacteria including *pneumococci* [14-17]. Among adults no such levels have been identified. Instead, it has been assumed that similar antibody concentrations are protective in adults as well. Given the variability of the various assays used by most of the major reference laboratories, it is reasonable to assume that long-term protection probably does result from a one-month post-vaccine concentration of between 1 and 1.5 mg/L [17]. However, which antibody levels would protect against infections may differ depending on subjects’ age, previous vaccination status, other medical conditions and/or concomitant immunosuppressive treatment [16].

After immunisation with pneumococcal conjugate vaccine in children protection was seen at lower post-vaccination antibody concentrations and antibody levels ≥0.35 mg/L were estimated to be associated with good protection against infections [18,19]. Studies investigating the associations between pre- and post-vaccination antibody levels and protection against infections after immunisation with pneumococcal conjugate vaccine in adult patients and with arthritis are lacking.

The aim of the present study was to explore the association between antibody levels before and after vaccination and the occurrence of pneumococcal infections up to 4.5 years before and after vaccination with 7-valent pneumococcal conjugate vaccine (PCV7) in patients with RA and SpA. In addition, the objective was to identify the antibody levels (cutoffs) associated with protection against putative severe pneumococcal infections. Finally, we wanted to study possible predictors of serious infections occurring after vaccination.

## Methods

### Patients

Adult patients with RA and SpA, including psoriatic arthritis, regularly followed at the outpatient rheumatology clinic, Skåne University Hospital in Lund and Malmö, Sweden were approached consecutively and invited to participate in the study as previously reported [20]. Eligibility criteria included no previous pneumococcal vaccination or vaccination with 23-valent pneumococcal polysaccharide vaccine ≥5 years before the study entry. Initially, 505 arthritis patients were enrolled. All participants were immunised with a single dose of 0.5 ml of PCV7 intramuscularly. Inclusion of patients and vaccination was performed over a time period of approximately 1 year (between May 2008 and June 2009).

An ethical approval, mandatory for the study, was received from the Regional Ethical Review Board in Lund, Sweden. Informed consent was obtained from all patients before inclusion in the study.

Antibody levels for two pneumococcal capsular polysaccharide antigens (6B and 23F) were measured before and 4 to 6 weeks after vaccination using enzyme-linked immunosorbent assay (ELISA) as previously reported [21]. The Skåne Healthcare Register (SHR) containing data on all in- and outpatient care in the region was used to search for serious pneumococcal infections using the International Classification of Diseases, tenth revision (ICD-10) coding system. All such events occurring between 31 December 2004 and 31 December 2012 were retrieved [13].

The following infections were included: pneumonia (J13.9, J18.0, J18.1, J18.9), lower respiratory tract infection (J22.9), septicaemia (A40.3), meningitis (G00.1) and septic arthritis (M002B, M002C, M002D, M002F, M002G, M002H, M002X, M00.1).

In order to reduce the risk of double documentation, we ignored all repeat codes within the same patient within 3 months from the first occurrence of the code. We performed validation of the diagnostic codes by scrutinising medical records of the patients identified with serious infections. A positive X-ray or blood culture, or a C-reactive protein ≥50 was defined as a confirmed event.

Of 505 initially immunised patients in total 497 patients (RA = 248 and SpA = 249) were included in the present study. The remaining eight patients were excluded due to moving from the Skåne region. All patients were divided into predefined treatment groups as previously described [20].

### Statistics

Pre-vaccination and post-vaccination geometric mean antibody levels (GML) for each serotype were calculated for patients with a history of serious infections and those without such infections separately. The Wilcoxon rank test was used to calculate the differences between post- and pre-vaccination antibody levels in patients with and without a history of serious infections, respectively. Differences in post-vaccination antibody levels between patients with and without serious infections were calculated using the Mann-Whitney *U* test. The impact of age, treatment and diagnosis on occurrence of serious infections after vaccination was analysed using a time-dependent Cox regression model and also binary logistic regression analysis. The association between post-vaccination antibody levels and infection after vaccination was explored using receiver operating characteristic (ROC) curves for each serotype separately. Youden’s index (J statistics) was calculated for all points of the ROC curves (J = specificity + sensitivity-1) and used as a criterion for selecting optimal cutoff level [22]. Antibody concentrations associated with protection against subsequent infections with high specificity and sufficient sensitivity were determined as cutoff levels. A two-tailed *P* value less than 0.05 was considered statistically significant.

## Results

Demographics, disease and treatment characteristics of the RA patients, the SpA patients and the study population as a whole, are summarised in Table 1. Patients with RA were in general older and the majority of these patients were women (81% versus 45% of SpA patients). At vaccination, 69% of RA patients and 33% of SpA were treated with methotrexate (MTX). In total, 34% SpA patients were on non-steroidal anti-inflammatory drugs (NSAIDs) only, while no RA patient received these remedies without MTX or tumour necrosis factor (TNF)-inhibitors. Approximately one-third of all patients were treated with anti-TNF + MTX and anti-TNF as monotherapy, respectively. The proportion of patients receiving biologic treatment did not differ between RA and SpA.

**Table 1:** **Demographic, disease and treatment characteristics of the study population**

	**All patients (n = 497)**	**RA patients (n = 248)**	**SpA patients (n = 249)**
Age, mean (SD) (range) years	55.7 (13.0) (22–86)	60.8 (12.4) (24–86)	50.6 (11.6) (22–76)
Sex (% women)	62.8	81.0	44.6
Smoking at vaccination (%)	17.4	19.0	15.7
Disease duration mean (SD) (range) years	14.9 (11.2) (0–48)	15.9 (11.5) (0–48)	13.8 (10.8) (0–45)
DAS28 (SD) (range)	3.2 (1.2) (0–6.4)	3.6 (1.1) (0.6-5.9)	2.8 (1.2) (0–6.4)
DAS28 CRP at vaccination (0–10)	2.7 (0.97) (0.96-5.6)	2.9 (0.9) (0.96-4.9)	2.5 (0.98) (0.96-5.6)
HAQ at vaccination (0–3) (range)	0.7 (0.6) (0–3.0)	0.9 (0.7) (0–3.0)	0.5 (0.5) (0–2.13)
RF at vaccination (%)	----	79.8	----
Anti-CCP at vaccination (%)	----	77.8	----
HLA-B27 (%)	----	----	47.8
MTX at vaccination (%)	51.1	69.4	33.0
MTX + anti-TNF at vaccination (%)	31.8	35.1	32.9
Anti-TNF as monotherapy (%)	34.0	30.6	32.9
NSAIDs without other anti-rheumatic treatment (%)	17.1	0	34.1
Serious infection before vaccination (number of events)	18	13	5
Serious infection after vaccination (number of events)	27	23	4

RA, rheumatoid arthritis; SpA, spondylarthropathy; DAS28, disease activity score using 28 joint counts; CRP, C-reactive protein; HAQ, health assessment questionnaire; RF, rheumatoid factor; anti-CCP, anti-cyclic citrullinated peptide; HLA-B27, human leukocyte antigen B27; MTX, methotrexate; anti-TNF, anti-tumor necrosis factor; NSAIDs, non-steroidal anti-inflammatory drugs.

In total, 83% of the infections before and 89% of the infections after vaccination could be confirmed in the medical records. Only one patient with a history of serious infections before vaccination (one RA patient with three episodes of pneumonia) was recorded for one additional serious infection after vaccination (pneumonia).

### Before vaccination

Eighteen serious infections occurred in 15 patients between 31 December 2004 and May 2008 (one patient had two and one additional patient had three infections), Table 1. Out of these 18 infections, 13 occurred among patients with RA and five among patients with SpA (of which two were in SpA patients on NSAIDs without disease-modifying anti-rheumatic drugs (DMARDs)).

The patients with a history of infections before vaccination had numerically but not statistically lower pre-vaccination antibody levels. Antibody levels increased significantly for both serotypes after vaccination among patients with, as well as without, infections before vaccination. (*P* <0.001 to 0.005 for 6B and *P* <0.001 to 0.01 for 23F).

### After vaccination

During the 4.5 years after vaccination, that is between May 2008 and 31 December 2012, 27 serious infections were identified in 23 patients (four patients had two infections), Table 1. Out of these 27 infections, 23 occurred in RA patients and four in SpA patients (of which only one in SpA patients on NSAIDs without DMARDs). One patient with three serious infections before vaccination (three events of pneumonia) had one recurrent infection after vaccination (pneumonia). Another patient with two serious infections before vaccination did not experience additional infections after vaccination. No other patients with a history of infections before vaccination had any serious infection after vaccination.

The patients with a history of serious infections after vaccination had lower pre-vaccination antibody levels for serotype 6B (*P* = 0.07) and 23F (*P* = 0.03) and lower post-vaccination antibody levels for both serotypes (*P* = 0.04 and *P* = 0.038 for 6B and 23F, respectively; Mann-Whitney *U* test), Table 2.

**Table 2:** **Geometric mean levels (GML) of pre- and post-vaccination antibody levels in patients with and without a history of serious infections after vaccination in all patients, and RA and SpA patients respectively**

**All patients**	**No serious infections after vaccination**	**Serious infections after vaccination**
	**N = 470**	**N = 27**
	**Antibody levels (mg/L)**	**Antibody levels (mg/L)**
	**GML; 95% CI**	**GML; 95% CI**
*Serotype 6B*		
Pre-vaccination	1.8 (1.6-2.1)	1.1 (0.6-1.9)
Post-vaccination	**4.1 (3.5-4.8)**	**2.2 (1.3-3.8)**
*Serotype 23 F*		
Pre-vaccination	0.8 (0.7-0.9)	0.4 (0.2-0.7)
Post-vaccination	**2.6 (2.2-3.0)**	**1.5 (0.9-2.3)**
**RA patients**	**No serious infections after vaccination**	**Serious infections after vaccination**
*Serotype 6B*		
Pre-vaccination	1.7 (1.4-2.1)	1.0 (0.5-1.8)
Post-vaccination	3.1 (2.5-3.9)	2.0 ( 1.1-3.7)
*Serotype 23 F*		
Pre-vaccination	0.7 (0.6-0.8)	0.3 (0.2-0.7)
Post-vaccination	1.8 (1.4-2.1)	1.4 (0.8-2.4)
**SpA patients**	**No serious infections after vaccination**	**Serious infections after vaccination**
*Serotype 6B*		
Pre-vaccination	1.9 (1.6-2.4)	2.2 (0.5-9.8)
Post-vaccination	5.2 (4.1-6.5)	3.6 (0.7-17.4)
*Serotype 23 F*		
Pre-vaccination	0.8 (0.7-1.0)	0.7 (0.2-2.3)
Post-vaccination	3.6 (3.0-4.5)	1.9 (0.5-6.9)

CI, confidence interval; RA, rheumatoid arthritis; SpA, spondylarthropathy.

### Cutoff levels

**Figure 1:**
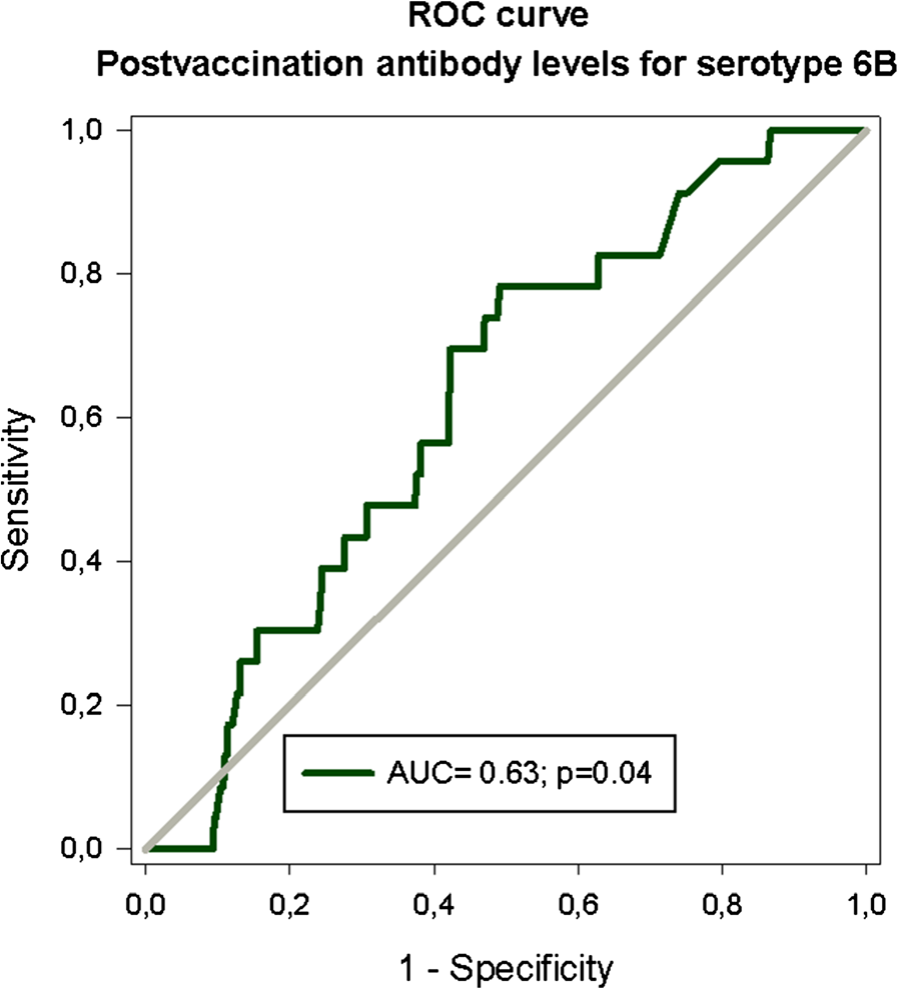
Receiver operating characteristic curve shows the association between post-vaccination antibody concentrations for serotype 6B and severe infections after vaccination. The rectangle indicates specificity and sensitivity levels chosen for determination of cutoff.

**Figure 2:**
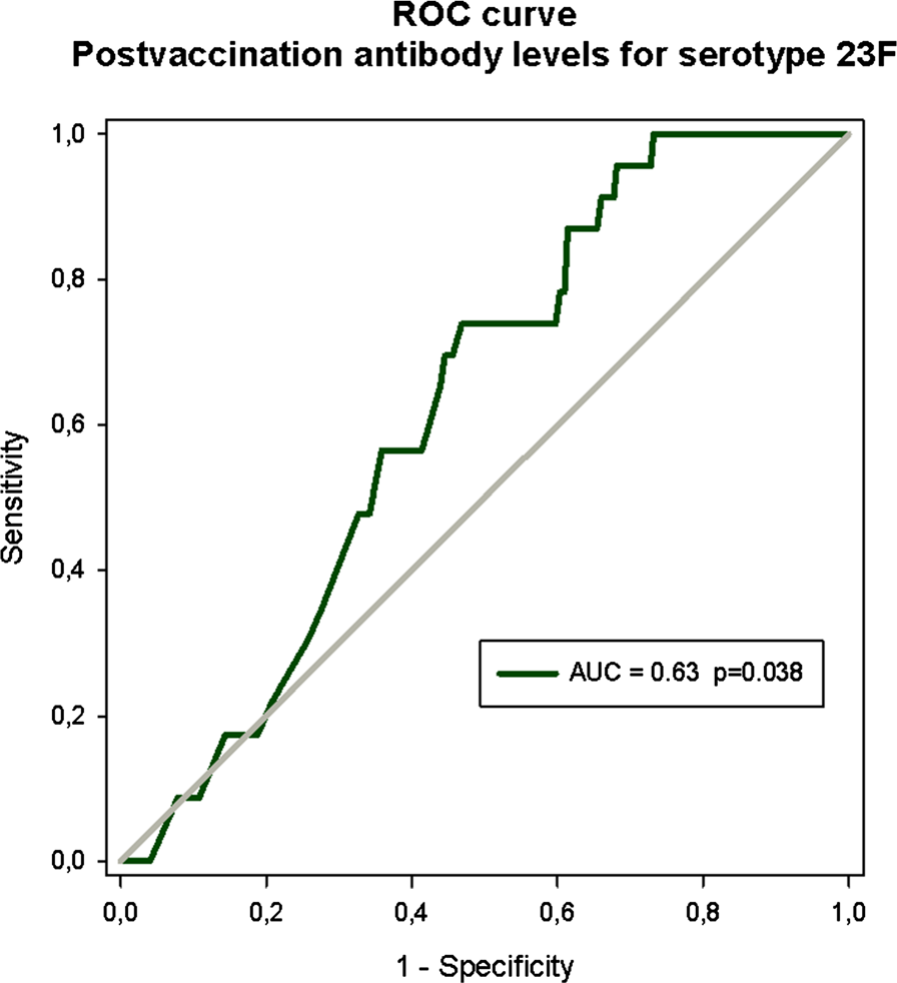
Receiver operating characteristic curve shows the association between post-vaccination antibody concentrations for serotype 23F and severe infections after vaccination. The rectangle indicates specificity and sensitivity levels chosen for determination of cutoff.

A post-vaccination antibody level of 1.29 mg/L for serotype 6B was identified as the most optimal cutoff level (75% specificity and 39% sensitivity; Figure 1). The corresponding cutoff levels for serotype 23F was 1.01 mg/L (73% specificity and 35% sensitivity; Figure 2) [22]. Table 3 shows in boldface the chosen cutoff levels for both serotypes. The cutoff for 6B could have been chosen at an antibody level of about 0.70 mg/L, since that is where the ROC curve indicates a deviation from the random of 45^○^, favouring the beginning of protection against infections, but the sensitivity was as low as 26%. For 23F, the beginning of deviation from the random of 45^○^ coincided more closely with acceptable sensitivity levels at antibody levels of about 1.01 mg/L.

**Table 3:** **Different post-vaccination concentrations for serotype 6B and 23F (cutoffs), corresponding specificity and sensitivity and predictive values in relation to occurrence of serious infections after vaccination identified using receiver operating characteristic (ROC) curves**

**Serotype 6B**			
**Post-vaccination antibody levels (mg/L)**	**Specificity (%)**	**Sensitivity (%)**	**Positive predictive value % (95% CI)**
0.20	95.1	0	0.0 (0–80.7)
0.44	90.3	4.35	2.1 (0.4-11.3)
0.69	85.1	26.1	7.8 (2.9-16.2)
1.02	79.1	30.4	7.0 (2.9-13.9)
**1.29**	**75.1**	**39.1**	**7.0 (3.3-12.9)**
1.31	74.9	39.1	7.1 (3.3-13.0)
1.45	73.0	39.1	6.6 (3.1-12.2)
1.51	72.4	43.5	6.4 (3.0-11.9)
1.65	70.3	43.5	6.6 (3.2-11.8)
**Serotype 23 F**			
**Post-vaccination antibody levels (mg/L)**	**Specificity (%)**	**Sensitivity (%)**	**Positive predictive value % (95% CI)**
0.18	95.1	8.7	0.0 (0–17.8)
0.46	85.0	17.4	5.6 (1.8-13.6)
0.71	80.0	21.7	4.9 (1.6-11.0)
0.88	75.3	26.1	5.2 (1.9-10.9)
**1.01**	**73.0**	**34.8**	**5.9 (2.6-11.3)**
1.10	71.3	34.8	5.6 (2.4-10.7)
1.11	71.1	39.1	6.5 (3.0-11.9)
1.22	69.6	43.5	6.4 (3.1-11.4)

CI, confidence interval.

### Predictors of serious infections

Table 4 summarises results of uni- and multivariate regression analysis using Cox regression model with regard to time between vaccination and occurrence of infection. Patients with serious infections after vaccination were older and they suffered more often from RA than SpA, but the 28 swollen and tender joint count, 66/68 swollen and tender joint count, disease activity score using 28 joint counts (DAS28) C-reactive protein (CRP) or health assessment questionnaire (HAQ) at vaccination did not differ between patients with or without such infections.

**Table 4:** **Predictors of serious infections using Cox regression analysis**

	***P*** **value**	**Odds ratio**	**95% CI**
**Univariate analysis**			
**Age (per 10 years)**	**<0.001**	**1.9**	**1.3-2.8**
Gender (female/male)	0.813	0.9	0.4-2.1
Disease duration at vaccination (years)	0.075	1.03	1.0-1.1
DAS28 at vaccination (0–10)^*^	0.366	1.2	0.8-1.7
HAQ at vaccination (0–3)^**^	0.332	1.4	0.7-2.6
**Diagnosis (RA/SpA)**	**0.004**	**4.8**	**1.7-14.2**
Methotrexate at vaccination (yes/no)	0.737	0.9	0.4-2.0
Anti-TNF at vaccination (yes/no)	0.380	1.5	0.6-3.9
Methotrexate + anti-TNF at vaccination (yes/no)	0.428	0.7	0.3-1.7
**Prednisolone at vaccination (yes/no)**	**<0.001**	**7.1**	**2.7-18.7**
**Prednisolone at vaccination (mg/day)**	**<0.001**	**1.2**	**1.1-1.3**
Smoking at vaccination (yes/no)	0.289	1.7	0.7-4.2
**Multivariate analysis**			
**Age (years)**	**0.034**	**1.5**	**1.0-2.2**
^**#**^Diagnosis (RA/SpA)	0.064	2.9	0.9-9.4
**Prednisolone at vaccination (yes/no)**	**<0.001**	**5.6**	**2.1-15.1**
**Prednisolone dose (mg/day)**	**<0.001**	**1.2**	**1.1-1.3**

^*^DAS28, disease activity score using 28 joint counts; ^**^HAQ, health assessment questionnaire; ^#^diagnosis (RA/Spa), prednisolone at vaccination (yes/no) and prednisolone dose (mg/day) are analysed in different regression models adjusted for age. CI, confidence interval; RA, rheumatoid arthritis; SpA, spondylarthropathy; anti-TNF, anti-tumour necrosis factor.

Patients with serious infections after vaccination received oral prednisolone to a larger extent. Mean daily prednisolone dose (range) in patients with and without history of serious infection was 3.8 (0 to 10) mg and 1.1 (0 to 20) mg, respectively. The majority of patients with serious infections were on higher prednisolone doses (fourth quartile that is ≥7.5 mg daily). Ongoing MTX, anti-TNF or combination of these treatments at vaccination were not associated with serious infections after vaccination, and there were no significant differences in disease duration at vaccination or between men and women. Smokers had numerically more infections but the difference was not significant.

When multivariate analysis was applied higher age remained a significant predictor of serious infections while differences between RA and SpA became borderline significant (*P* = 0.064). Prednisolone treatment at vaccination (yes/no) remained a statistically significant predictor of serious infections after adjustment for age (*P* <0.001) as well as higher prednisolone doses (*P* <0.001). After adjustment for age, neither MTX, biological treatment nor combination of these at vaccination predicted serious infections. The limited number of events (that is infections) precluded adjustment for other disease or treatment characteristics. Similar figures were found when using an adjusted binary logistic regression model.

## Discussion

In the present study, we report the association between post-vaccination levels of antibodies to pneumococcal capsular polysaccharides and protection against subsequent putative serious pneumococcal infections. Moreover, we identified suggestive post-vaccination cutoff antibody levels associated with the most optimal combination of sensitivity and specificity for such infections to be used for further research.

Using ROC, we identified post-vaccination antibody levels, which we consider to have sufficiently high specificity but not too low sensitivity to be protective against infections. Interestingly, these levels were rather close to levels of 1 mg/L extrapolated from previous studies investigating antibody response after *Hib* vaccination using polysaccharide vaccine in children and which have subsequently been generally accepted [14,15,17]. Antibody response and protective levels after vaccination with more immunogenic conjugate vaccine are not established in adults and may differ between different serotypes. However, we previously reported similar antibody levels after vaccination with PPV or PVC in patients with RA receiving MTX, anti-TNF or combination of these treatments [23].

In all patients post-vaccination antibody levels increased significantly, although patients experiencing serious infections overall had lower levels compared to patients without serious infections.

Patients with a history of serious pneumococcal infection within 4 years before vaccination had somewhat lower pre-vaccination antibody levels than patients without such infections although the difference was not statistically significant. Pre-vaccination antibody levels reflect the antibodies naturally acquired in response to infections and would be expected to be higher in patients who had such infections. Most infections (13 of 18) occurred in RA patients receiving immunosuppressive treatment (MTX, anti-TNF or combination of these) when blood samples were taken (immediately before vaccination). These remedies could have diminished pre-existing antibody levels, which is in line with our previous study where both MTX anti-TNF were associated with low persistence of protective immunity 1.5 years after pneumococcal vaccination in RA [24]. Unfortunately, no control group was included in the study and we were unable to measure antibody levels in the non-vaccinated epidemiologically matched control population and, therefore, could not estimate the impact of immunosuppression on these levels.

As expected, older patients and those with RA had more serious infections after vaccination but only age remained a significant predictor after adjustment for diagnosis. Of anti-rheumatic treatments only ongoing prednisolone remained a strong predictor after adjustment for age. This is in agreement with previous reports on association between increasing age and prednisolone treatment and infections in RA [25-27]. Interestingly, higher prednisolone dosage was associated with increased infection risk even with the relatively moderate prednisolone doses in the current study (max 10 mg/day). One of our inclusion criteria was unchanged doses of anti-rheumatic drugs (including prednisolone) for at least 4 weeks before study entry [20]. This criterion possibly/probably reflects a group of patients in need of an overall larger long-term prednisolone exposure. The association between low/moderate prednisolone doses >1 month and increased risk of serious infections support the latest European League Against Rheumatism (EULAR) guidelines for the management of new-onset RA recommending the tapering of prednisolone as fast as clinically feasible [26]. Treatment with MTX, anti-TNF or both combined did not contribute to risk of serious pneumococcal infections in accordance with other reports [25,27].

Compared to RA, fewer SpA patients experienced serious infections before and also after vaccination. SpA patients as a group were younger and one-third was treated with analgesics/NDSAIDs without any DMARDs, which is a probable explanation for the difference. This is supported by the disappearance of the difference between RA and SpA after adjustment for age in the multivariate analysis.

The present study has important limitations. Usually, two serological methods are used to estimate the protective antibody levels: standard ELISA measuring antibody concentrations and opsonophagocytic assays measuring the functionality of the antibodies. Unfortunately, the opsonophagocytic assay was not available at our laboratory. On the other hand, a fair correlation between antibody concentrations and their opsonophagocytic capacity has been shown (r between 0.72 and 0.91) [16,28].

Infections were identified from the healthcare register using ICD-10 diagnostic codes for known putative pneumococcal infections. However, when medical records of patients diagnosed with putative serious pneumococcal infections were scrutinised, we found that the vast majority of these infections (83% of serious infections before and 89% after vaccination) were based on X-ray findings or laboratory tests. This supports the bacterial origin of the infections but does not prove that infections were caused by *pneumococci.* On the other hand, *Streptococcus pneumoniae* is known to be the most common cause of community-acquired pneumonia in adults [29]. Serotypes included in the 7-valent conjugate vaccine are shown to account for 50 to 65% of invasive pneumococcal diseases in adults >50 years of age [29,30]. Furthermore, we compared the numbers of infections before and after an intervention in the same cohort using the same diagnostic codes and thus the proportion of potentially misclassified events is expected to affect pre- and post-vaccination in a similar way.

In the present study, we chose to measure antibody response to only two of seven different serotypes (6B and 23) presented in the vaccine. These serotypes are included in both PPV and pneumococcal conjugate vaccines currently available on the market and have been reported to be associated with severe infections and serious clinical outcome among adults [31]. Although the putative protective levels probably differ between the serotypes, we assume that immunosuppressive treatments would influence the antibody response to all serotypes in the same manner.

Another weakness is that the number of events was limited, which precluded analysis of disease and treatment characteristics or co-morbidities on the occurrence of infections. In addition, when the study was initiated we did not have the possibility to include a control group of patients or healthy controls. In spite of these limitations, the present study is the first to report on the association between antibody response and protection against infections after immunisation with pneumococcal conjugate vaccine in adult immunosuppressed patients with arthritis. Furthermore, we confirmed that putative protective antibody levels used for healthy adults are applicable for arthritis patients receiving modern anti-rheumatic treatments including MTX, anti-TNF remedies and their combination.

The vast majority of serious infections occurred in patients receiving immunosuppressive treatment. We have reported diminished antibody response in RA patients from this cohort treated with MTX and to a lesser extent in arthritis patients on anti-TNF [11]. We also recently demonstrated that these patients had limited protection against serious infections after pneumococcal vaccination [13]. In order to induce better antibody response and thus better protection against infections these findings indicate that pneumococcal vaccination should preferably be given before the initiation of immunosuppressive treatment. As shown by Desai *et al*. measuring pneumococcal vaccination rates in a rheumatology practice and improving those rates is important for encouraging rheumatologists to vaccinate their patients before starting immunosuppressive treatment [32,33].

## Conclusions

In summary, measurement of antibody response after immunisation with a single dose of pneumococcal conjugate vaccine using standard ELISA is a feasible method for estimating protection induced by vaccination. Approximately a month after immunisation, antibody levels ≥1.01 to 1.29 mg/L against serotypes included in the vaccine are associated with protection against severe putative pneumococcal infections in patients with arthritis treated by modern anti-rheumatic treatments.
